# Test methods for surface disinfection: comparison of the Wiperator ASTM standard E2967-15 and the 4-field test EN 16615

**DOI:** 10.3205/dgkh000339

**Published:** 2020-04-01

**Authors:** Anja Jacobshagen, Stefanie Gemein, Martin Exner, Jürgen Gebel

**Affiliations:** 1 Institute for Hygiene and Public Health, University Hospital Bonn, Bonn, Germany; 2 VAH c/o Institute for Hygiene and Public Health, University Hospital Bonn AöR, Bonn, Germany

**Keywords:** surface disinfection, Wiperator, 4-field test, disinfectant wipe

## Abstract

**Aim:** Two test methods for surface disinfection (phase 2, step 2) – the Wiperator method (ASTM standard E2967-15) and the 4-field test (EN 16615) – were compared using a disinfectant solution based on quaternary ammonium compounds and a ready-to-use alcohol-based wipe. As test organisms, *Staphylococcus* *aureus* and *Pseudomonas* *aeruginosa* were used.

**Results:** While the 4-field test is a manual method and better reflects the process in practice, with the Wiperator, the wiping process is better controlled because it is an automated procedure. A comparison of the effects of both methods on the target log_10_-reduction of *S. aureus* and *P. aeruginosa* indicates a statistically significant difference between the two test methods (Mann-Whitney *U*-Test. *S. aureus*: 0 (U_min_)<4 (U*_crit_*); *n*_1_=8, *n*_2_=8, *p*=0.001; 2-sided. *P. aeruginosa*: 24 (U_min_)<26 (U_crit_); *n*_1_=11, *n*_2_=10, *p*=0.025, 2-sided). In addition, the results indicate that the wipe used has a major influence on the success of the disinfection process.

**Discussion:** Both methods are suitable for efficacy studies of surface disinfectants, yet they differ in some aspects. Additionally our data indicate a statistically significant difference between the two test methods.

**Conclusion:** Efficiency testing of surface disinfection is a complex process that depends on many different parameters. Since the 4-field test better reflects the practice, it makes sense to stick to this test procedure, taking into account that the EN 16615 was approved by CEN TC 216 in 2015 after method validation ring trials.

## Zusammenfassung

**Ziel:** Zwei praxisnahe Testmethoden (Phase 2, Stufe 2) für die Flächendesinfektion – die Wiperator-Methode (ASTM-Standard E2967-15) und der 4-Felder-Test (EN 16615) – wurden verglichen. Als Prüfprodukte wurden eine Desinfektionslösung auf Basis von quartären Ammoniumverbindungen und ein gebrauchsfertiges Desinfektionstuch auf Basis von Alkoholen verwendet.

**Ergebnisse:** Während es sich beim 4-Felder-Test um eine manuelle Methode handelt, die die Praxis besser widerspiegelt, ist die Wiperator-Methode ein maschinelles Verfahren mit einem kontrollierteren Wischvorgang. Im Vergleich der Wirkungen beider Verfahren auf die Zielgröße log_10_-Reduktion von *S. aureus* und *P. aeruginosa* anhand zweier unabhängiger Stichproben ergab sich zwischen beiden Testverfahren ein statistisch signifikanter Unterschied (Mann-Whitney *U*-Test: *S. aureus*: 0 (U_min_)<4 (U_krit_); *n*_1_=8, *n*_2_=8, *p*=0,001; 2-seitig. *P. aeruginosa*: 24 (U_min_)<26 (U_krit_); *n*_1_=11, *n*_2_=10, *p*=0,025; 2-seitig. Die Ergebnisse zeigen ferner, dass das verwendete Tuch einen Einfluss auf den Erfolg des Desinfektionsprozesses hat.

**Diskussion:** Der 4-Felder-Test und die Wiperator Methode sind standardisierte Verfahren und eignen sich für Wirksamkeitsuntersuchungen von Flächendesinfektionsmitteln, unterscheiden sich aber in einigen Aspekten: Verfahrensart (manuell – automatisiert), Wischvorgang (horizontal – punkförmig kreisend), Standardtuch (SCA-Wipe – J-cloth), Dauer des Wischvorgangs, Größe des Inokulums und Testfläche. Zusätzlich zeigen unsere Daten einen statistisch signifikanten Unterschied zwischen den beiden Testmethoden auf.

**Schlussfolgerung:** Die Wirksamkeitsprüfung von Flächendesinfektionsmitteln ist ein komplexer Prozess, der von verschiedenen Parametern abhängt. Da der 4-Felder Test die Einflussfaktoren der praktischen Anwendung im Testdesign berücksichtigt, ist es sinnvoll, an diesem Prüfverfahren festzuhalten. Zudem wurde der 4-Felder Test vom CEN TC 216 im Jahr 2015 nach Ringversuchen zur Methodenvalidierung als EN 16615 genehmigt.

## Introduction

Surfaces in the patient area can harbor many microorganisms and represent a reservoir for pathogens [1], [2], [3]. Many studies have demonstrated the risk of cross-transmission of pathogens [4], [5]. Surface disinfection is an important measure in hospital hygiene strategy to prevent the spread of infections with nosocomial pathogens in hospitals [6]. In 2004, the Commission for Hospital Hygiene and Infection Prevention (KRINKO) published the guideline “Requirements for hygiene in the cleaning and disinfection of surfaces” [7]. Various studies have shown the effectiveness of surface cleaning and disinfecting procedures [8], [9]. In addition, different test methods have been discussed [3]. However, methods to test the effectiveness of surface disinfectants under practical conditions (Phase 2, Step 2 of the European test hirarchy) are few. Today, two of the methods have been established as standards: In Europe it is the 4-field test (EN 16615) [10] and in the US it is the Wiperator ASTM Standard E2967-15 [11]. Both methods allow the evaluation of removal, inactivation and transfer of test organisms from a test surface by using disinfectant wipes as offered by the manufacturer. Sattar et al. [12] examined and compared the 4-field test with the Wiperator ASTM standard E2967-15. The authors named the “uncontrollability of the wiping process” as the weak point of the 4-field test, which, according to the authors, can be attributed to the manual part of the method. In contrast, the Wiperator method would allow a controlled wiping movement. 

In addition to a theoretical comparison of the 4-field test and the Wiperator method, the subject of the study presented here was to apply both methods under practical conditions. A surface disinfectant solution based on quaternary ammonium compounds and an alcohol-based ready-to-use wipe (rtu-wipe) were employed for this purpose. As test organisms, *Stapylococcus* *aureus* and *Pseudomonas* *aeruginosa* were used. The aim was to evaluate the two methods by measuring the bactericidal activity of the tested products to determine whether there is a statistically significant difference between the methods, and by examining the practical relevance of the methods.

## Materials and Methods

### Disinfectants

A disinfectant solution based on quaternary ammonium compound (QAC) was used at 1% and 2%; its active ingredients in 100 g were 6 g of didecyldimethylammonium chloride (CAS Nr. 7173-51-5) and 5.5 g of N-(3-aminopropyl)-N-dodecylpropan-1,3-diamine (CAS Nr. 2372-82-9) (B. Braun Melsungen AG, Germany). This was compared with a pre-wet alcohol-based rtu-wipe that comes in a soft pack of 90 wipes; its active ingredient in 100 g comprised 30 g of propan-2-ol (CAS Nr. 67-63-0) and 30 g of propan-1-ol (CAS Nr. 71-23-8) (Schülke & Mayr GmbH, Norderstedt, Germany). The first three wipes of the sealed package were discarded before performing the test run. 

### Wiping cloths

The standard cloth for the 4-field test (SCA-wipe) consists of 55% cellulose and 45% polyethylenterephtalate (TORK, Essity Professional Hygiene GmbH, Mannheim, Germany). The standard cloth for the Wiperator method (J-cloth) consists of viscose (art. no. JJ 30481 Johnson & Johnson, distributer E.D. Smith Foods Ltd. Ontario, Canada).

### Test surfaces

For the 4-field test, polyvinyl chloride (PVC) rectangles (20 cm x 50 cm, 2 mm thickness) coated with PUR (polyurethane, art.no. 521-029, solid pur 2.0) were used (Lotter & Liebherr GmbH, Bonn, Germany). For the Wiperator method, disks of stainless steel, diameter 10 mm, 0.74 mm thickness, weighing 0.45 g) were employed (FILTAFLEX Ltd, Almonte Ontario, Canada, [13]). 

### Bacterial strain, medium and growth conditions

*Staphylococcus* *aureus* ATCC 6538 and *Pseudomonas* *aeruginosa* ATCC 15442 were chosen as Gram positive and Gram negative species, respectively. The initial suspension contained 1.5–5x10^9^ colony forming units (CFU) per ml. Strain maintenance, enrichment, cultivation, and detection of the test organisms were performed according to EN 12353 [14]. As the culture medium, Tryptone-Soya-Agar (TSA poured-plates) was used.

### Organic load

All tests were performed under clean conditions (0.03% albumin) following EN 16615 [10]. 

### 4-field test

**Figure 1:**
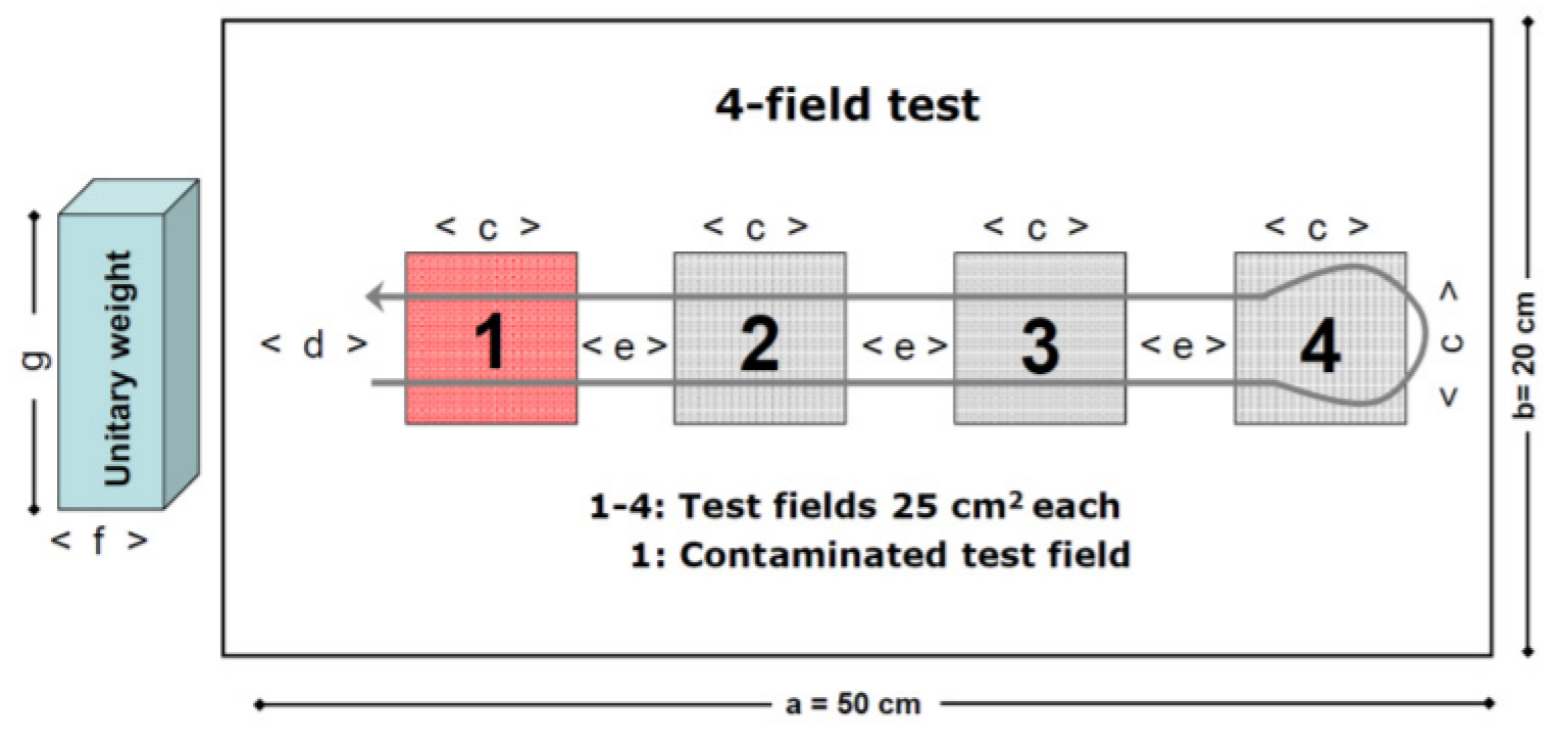
Diagram of the 4-field test [10] showing test surface (20x50 cm) with four test fields (5x5 cm) and stipulated wiping route of the wiping cloth. a=50 cm, b=20 cm, c=5 cm, d=10 cm, e=5 cm; the f and g dimensions of the unitary weight were at least 8.6 cmx12.1 cm, respectively, the block weighs 2.5 kg. The wiped area includes fields 1–4 with the turnaround at test field 4. Test field one is contaminated [10].

EN 16615 [10] was followed. The schematic procedure is outlined in Figure 1 (Fig. 1).

Prior to wiping, the SCA-wipe was soaked with 16 ml of disinfectant solution for 30 min; the J-cloth was soaked with 20 ml. The criterion for the soaking volume was that the wipe should be completely saturated. The rtu-wipes were used immediately after opening. Each wet wipe was placed on a granite block (unitary weight) and the wiping was performed as shown in Figure 1 (Fig. 1). After the contact time (15 min using the disinfectant solution and 5 min using the rtu-wipe), the recovery of the test organisms from test fields 1–4 was determined with the cotton-swab method according to EN 16615 [10]. The applied volume on the surface was identified by determining the weight of the wipe before and after the wiping process.

### Wiperator method

**Figure 2:**
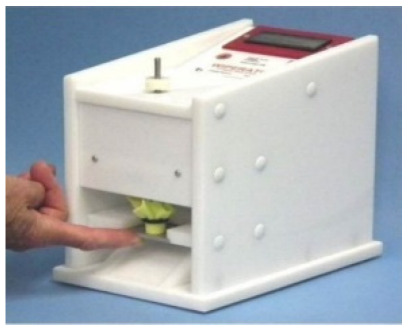
Wiperator [13]

The ASTM Standard E2967-15 was followed [11]. The setup is shown in Figure 2 (Fig. 2).

After pre-soaking the wipe with the disinfectant solution (840 µl, wipe in double, because the boss would not hold), the wiping process started on disk No. 1 for 10 seconds followed by wiping disk No. 2 for another 10 seconds with the same wipe. After the contact time (15 min using the disinfectant solution and 5 min using the rtu-wipe), the surviving cells were recovered by transferring the wiped disks into a vial containing 5 ml neutralization solution and glass beads. After the appropriate neutralizing time, the required dilution was spread on TSA plates. The used wipe was weighed before wiping disk No. 1 and after wiping disk No. 2. The difference in weight was calculated as the applied volume.

### Drying Control D_C0_ and D_Ct_ (valid for both methods)

In order to quantify the recoverability of CFU without any chemical or mechanical influence, two control-test surfaces á 5x5 cm (D_C0_ and D_Ct_) were contaminated on a separate test field parallel to the contamination of test field 1 for the 4-field test (in case of the Wiperator, this was a separate control disk). The recovery of test field D_C0_ took place immediately after drying and before wiping the contaminated test surfaces. The test organisms from test field D_Ct_ were recovered after the contact time (t) to quantify whether the test organisms were inactivated during the contact time without treatment. 

### Water Control

Water of standardized hardness (WSH) additionly containing 0.1% polysorbate 80 was used as a control. The final hardness was 375 ppm calculated for CaCO_3_; the pH was 7.0±0.2. The products were neutralized with a suitable neutralizer (TSHC: 30 g/l polysorbate 80, 3 g/lecithin, 1 g/l L-cysteine ad 1000 ml Trypton-NaCl). To determine the number of CFU per 25 cm^2^ without test product exposure, contaminated areas were treated with WSH. 

### Calculation of log_10_ reduction

 CFU were set in relation to the number of CFU of the untreated control field DCt. The results were converted into logarithmic_10_ values, defined as log_10_-reduction (R). 

4-field test: log_10_ reduction=log_10_ (CFU_DCt_)–log_10_ (CFU_T1_), whereas CFU_DCt_ is the number of CFU per 25 cm^2^ on control field D_Ct_ and CFU_T1_ corresponds to the number of CFU per 25 cm^2^ on test field 1. For the 4-field test, a log_10_ reduction of ≥5.0 was regarded as adequate bactericidal activity. 

Wiperator method: log_10_ reduction=log_10_ (CFU_DCt_)–log_10_ (CFU_D1_), CFU_DCt_ is the number of CFU per ml on control disk D_Ct_x5 and CFU_D1_ is the number of CFU per ml on disk no. 1x5. For the Wiperator method, a log_10_ reduction of >4.0 was regarded as adequate bactericidal activity [15].

### Liquid realease

In order to estimate the released volume, each wipe was weighed before and after wiping for each method. 

### Statistical analysis

 The data is the result of at least three replicates. For log_10_ reduction mean value (MV) and standard deviation (SD) were calculated. 

In order to determine whether there was a statistically significant difference between the 4-field test and the Wiperator method, the means of the log_10_-reduction of *S. aureus* after exposure to WSH with SCA-wipe (5 and 15 min) of both methods were compared using the Mann-Whitney-*U*-test (two independent samples, 2-sided, *p*=0.001 (*S. aureus*) and *p*=0.025 (*P. aeruginosa*). All analyses were completed in Excel 2016.

## Results

### Comparison of methods

In Table 1 (Tab. 1), major procedural aspects of the 4-field test and the Wiperator method are compared. Both methods are standardized procedures which differ in many ways. Major differences exist in the method (manual versus automated) and in the type of movement (straight across the surface versus circular on the spot). 

### Recovery rate

The recovery of the bacteria from the test surface is a fundamental challenge for both methods. Table 2 (Tab. 2) gives an overview of this data. The density of the test suspension of *S. aureus* and *P. aeruginosa* was within the range set by EN 16615 (9.17≤log_10_ N≤9.70). The range for D_C0_ and D_Ct_ for bactericidal efficacy is (6.88≤log_10_ N≤8.40) and was met for *S. aureus* but not *P. aeruginosa* with the 15-min contact time. There is a difference between the recovery of *P. aeruginosa* for D_Ct_ at 5 min and 15 min, especially in terms of the Wiperator results (6.9 log_10_–6.4 log_10_). This reveals the problem of loss of CFU of *P. aeruginosa* due to drying.

### Efficacy

The practical comparison included three efficacy studies with both methods using the surface disinfectant solution based on QAC at 2% and 15 min contact time with SCA-wipe. In a further experimental setup, the disinfectant solution was used at a reduced concentration of 1% and 15 min with SCA-wipe and J-cloth, in order to make differences between the methods more obvious. Finally, to cover the scope of the Wiperator, a ready-to-use, alcohol-based wipe was tested with both methods.

QAC at 2% (concentration-time relation as used in the European interlaboratory comparison) at 15 min exposure time was highly effective against *S. aureus* and *P. aeruginosa* in combination with the SCA-wipe with both methods (Table 3 (Tab. 3)). The log_10_ reduction was consistently >5 log_10_. The WSH control produced a greater reduction of CFU when the 4-field test was used (3.5–5.6). This is particularly noticeable for *P. aeruginosa* (5.6). The data of the drying control indicates that this might be due to the loss of CFU during the drying process. The mean log_10_ value of D_C0_ and D_Ct_ for *P. aeruginosa* was 6.7 and 6.3, resp., for the 4-field test, and 6.6 and 6.4, resp., for the Wiperator method (Table 2 (Tab. 2)). The loss of CFU may have a greater impact using 4-field test because this has more inoculum (50 µl) than does the Wiperator (10 µl), and thus drying time and stress for the cells is prolonged. 

QAC was effective at 1% and 15 min exposure using the SCA-wipe combined with both methods (Table 4 (Tab. 4)). Both methods showed good data reproducibility with regard to the standard deviation of values below 0.5. QAC was not effective at 1% and 15 min contact time using the J-cloth with the 4-field and with the Wiperator method. This is an indication that the wipe used had an influence on the bactericidal effect on QAC. Looking at the WSH-control, both methods showed a similar log_10_ reduction of CFU following wiping with the SCA-wipe and the J-cloth.

The alcohol-based rtu-wipe was not effective at 5 min exposure time for *S. aureus* and *P. aeruginosa* with the 4-field test (Table 5 (Tab. 5)). The log_10_ reduction was consistently <5.0. With the Wiperator method, however, the results were more differentiated: the alcohol-based rtu-wipe showed poor bactericidal activity against *S. aureus* with a log_10_ reduction of 2.5, but adequate bactericidal activity against *P. aeruginosa* with a log_10_ reduction of 5.0 log_10_. The better bactericidal effect against *P. aeruginosa* with the Wiperator method may be the result of CFU loss during drying process. 

### Mann-Whitney-U-Test

A comparison of the effects of both methods on the target log_10_-reduction of *S. aureus* and *P. aeruginosa* indicates a statistically significant difference between the two test methods (Mann-Whitney-*U*-Test. *S. aureus*: 0 (U_min_)<4 (U_crit_); *n*_1_=8, *n*^2^=8, *p*=0.001, 2-sided. *P. aeruginosa*: 24 (U_min_)<26 (U_crit_); *n*_1_=11, *n*_2_=10, *p*=0.025, 2-sided; Table 6 (Tab. 6)). 

### Liquid realease

For both SCA- and rtu-wipes, up to 6 times more volume was emitted with the Wiperator than with the 4-field test (Table 7 (Tab. 7)). For the J-cloth, the results of liquid release are very similar between the 4-field test and Wiperator. This indicates that the amount of liquid released by the wipe depends on the combination of method and wipe.

## Discussion

The environment in the patient’s proximity is a possible reservoir of pathogenic and potentially pathogenic microorganisms. There is evidence of the association between nosocomial infections by microorganisms and contaminated surfaces in hospitals [16], [17]. These microorganisms can survive for weeks or months on inanimate surfaces where they pose a risk for their spread [18]. Therefore, surface disinfection is an important prophylactic measure to prevent the spread of infections.

The 4-field test and the Wiperator method are two practicable procedures for testing the bactericidal activity of surface disinfectants (phase 2, step 2). The methods differ, however: the 4-field test is a manual procedure and the Wiperator method an automated procedure. Furthermore, the mode of wiping varies: In the 4-field test, the surface is wiped with a horizontal movement; with the Wiperator method, the surface is wiped in circles on one spot, which is a more dynamic process according to Edwards et al. [19]. Finally, the methods differ in the selection of the standard cloth, the duration of the wiping process, the pressure and kind of surface used, and in performance criteria. 

The 4-field test better reflects the process in practice due to the motion of wiping, the pressure applied, and the duration of the wiping process. The Wiperator, on the other hand, is more precise due to the automated wiping process, but the pressure applied during wiping process little resembles that applied in practice. This has also been stated by Kenters [20] who said that “*it* (the pressure of the Wiperator [note of the present authors]) *is not comparable with real-life situations*”. Nevertheless, it must be borne in mind that “*the closer the conditions are to practice, the more difficult it becomes to control variables*” as pointed out by Bloomfield et al. [21]. 

The EN 16615 was approved by CEN TC 216 in 2015 after method validation ring trials. On behalf of CEN TC 216 the Association for Applied Hygiene has carried out a further interlaboratory test according to 4-field test in 2018 [22]. Overall 12 laboratories participated in this ring trial according to EN 16615. The results showed a comparable log_10_ reduction, reproducibility and repeatability for 3 different biocidal formulations [22]. The control of variables is a topic always in the revision of EN standards and this also applies to this very new test procedure that has already proven itself.

With regard to comparability of the results generated by the two methods, the data indicated a statistically significant difference between the methods. In this context, it is important to mention that the combination of method and wipe seems to have a decisive influence on bactericidal activity. In terms of bactericidal activity of surface disinfectants, the present study indicates that the combination of wipe and method lead to different results. The disinfectant based on QAC at 1% was bactericidally effective in both methods only when combined with the SCA-wipe, not with the J-cloth. Overall, there is a tendency towards greater CFU reduction when the Wiperator method was used. This is probably due to the already mentioned parameters: high pressure, excessive volume-output, and longer application time. In addition, the dynamic wiping movement contributes to this effect. Edwards et al. [19] pointed out that during dynamic wiping, “*shear and compressive forces are applied*”. These forces help detach the bacteria from the surface and transfer them to the wipe. It seems evident that when moving the wipe in a horizontal action like in the 4-field test, these forces are not as strong [19]. 

Using *P. aeruginosa* as a test organism involves a decrease of CFU during drying. These results agree with a statement by Kenters [20], who said that “loss of drying can influence the test results”. From that point of view, the Wiperator method is preferable, because the inoculum is 5 times smaller, which reduces the drying time. However, our data do not show that the loss of CFU during drying was less with the Wiperator method. This must be investigated further.

Summing up, the 4-field test and the Wiperator method are suitable practical test methods for surface disinfection. Regarding the scope, the 4-field test considers different types of application of surface disinfectants (test product with non-specific wipe, pre-wet wipe-system in a container, ready-to-use wipes), whereas the Wiperator considers rtu-wipes only. In addition, the 4-field test evaluates the bactericidal effectiveness of surface disinfectants in a practical laboratory test. These factors are very important, because the reductions of CFU achieved in purely laboratory tests often cannot be achieved under real conditions with the same disinfectant [21]. Taking into account that the 4-field test better reflects actual practice and our data indicate a statistically significant difference between the methods, the 4-field test is clearly preferable, even though Kenters et al. stated “in order to generate reliable data and safe products both methods should be used” [20].

## Conclusion

The results demonstrate that efficiency testing of surface disinfection is a complex process that depends on different parameters. In previous test methods, only the disinfectant was tested. The two presented methods also consider the wipe and the amount of disinfectant solution used for one wipe. This is important, because presoaked wipe products and ready-to-use wipes are becoming more common in the hospital setting, thanks to high compliance of hospital personal with the use of these products [23], [24]. Generally speaking, automatic procedures are to be preferred due to their controlled action. However, they should not differ too much in their parameters from real conditions which speaks in favor of the 4-field test. Since the 4-field test better reflects the practice, it makes sense to stick to this method described in EN 16615.

It remains a challenge to find an automatic method that resembles surface disinfection in practice and at the same time provides proof of bactericidal activity.

## Notes

### Acknowledgements 

The authors thankfully acknowledge the consent of the concerned hospital to publish this work. Special thanks to Sylvia Koch and Stefanie Kowol for their constant support during testing procedure and Felix Waßer for the support during the statistical analysis.

### Competing interests

The authors declare that they have no competing interests.

## Figures and Tables

**Table 1:**
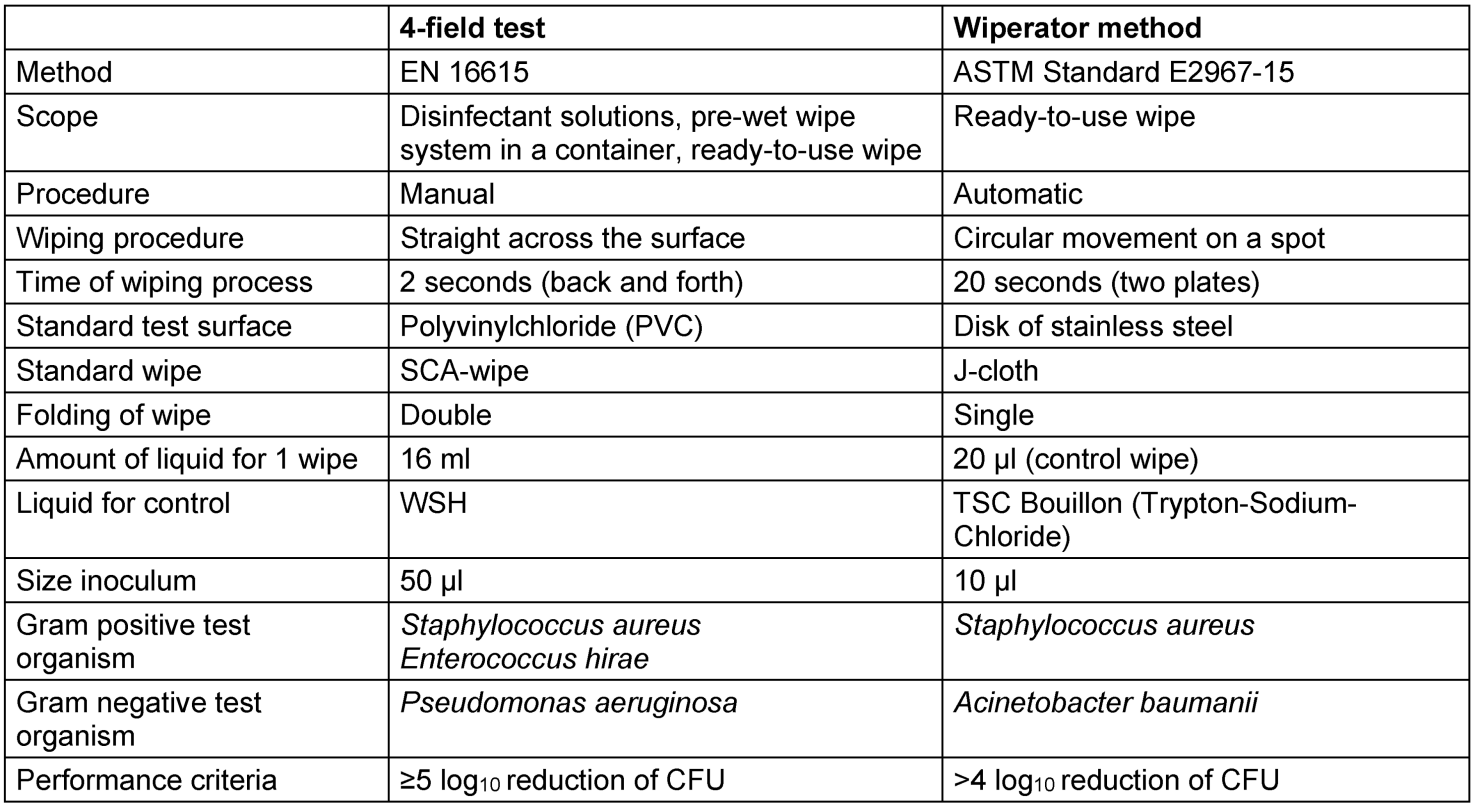
Comparison of 4-field test and Wiperator method

**Table 2:**
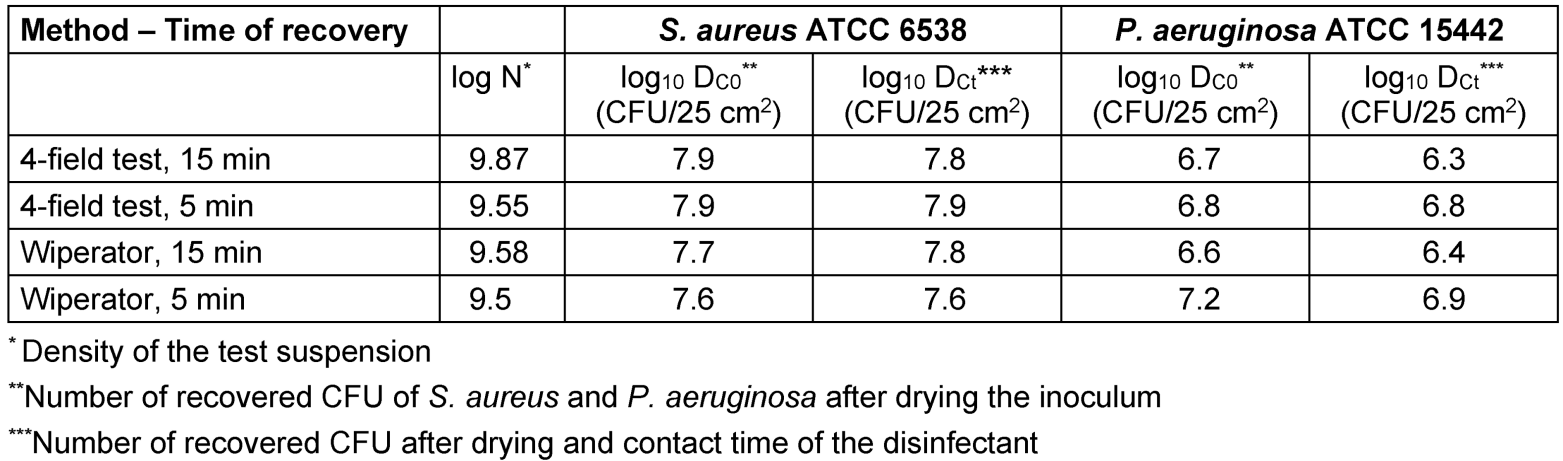
Test suspension and recovery of CFU of *S. aureus* and *P. aeruginosa* (*n*=3)

**Table 3:**
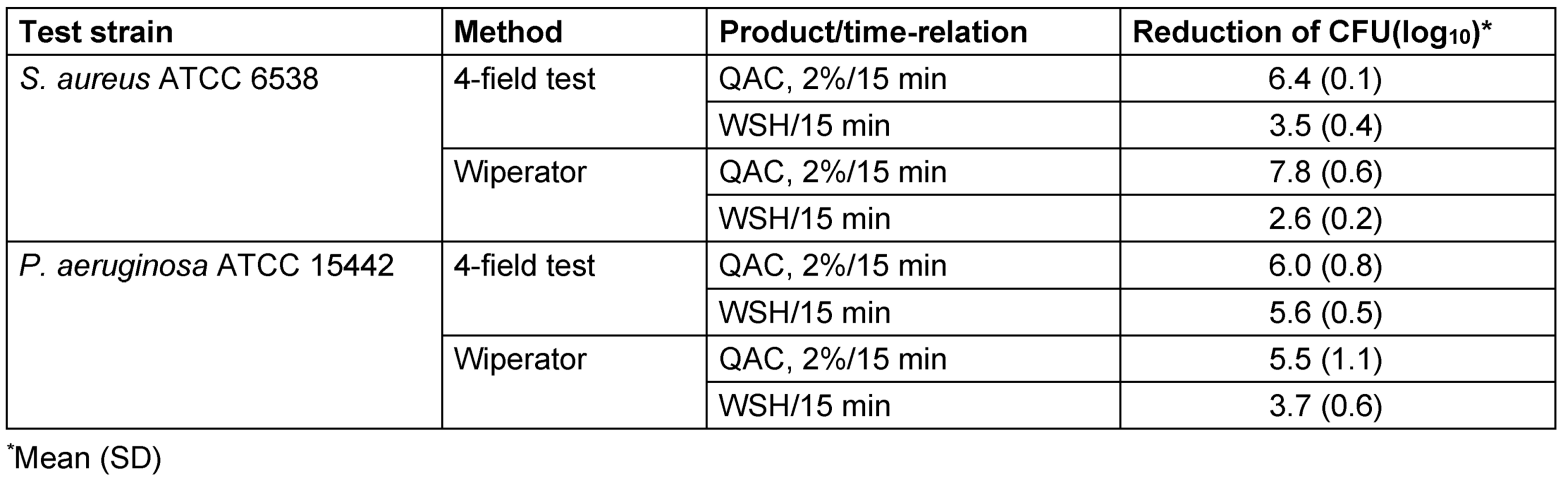
Mean log_10_ reduction of *S. aureus* and *P. aeruginosa* by exposure to a disinfectant solution based on QAC at 2% with a SCA-wipe with both the 4-field test and Wiperator method at a contact time of 15 min (*n*=3)

**Table 4:**
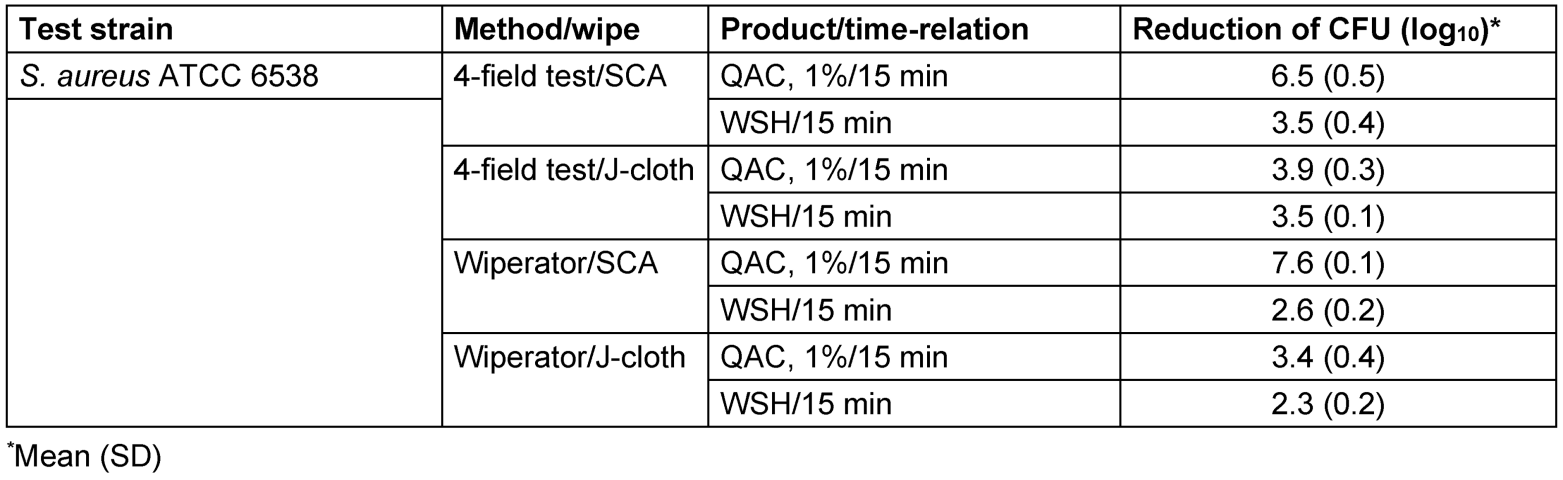
Mean log_10_ reduction of *S. aureus* by exposure to a disinfectant solution, QAC at 1%, 15 min contact time using SCA-wipe and J-cloth in combination with 4-field test and Wiperator method (*n*=3)

**Table 5:**
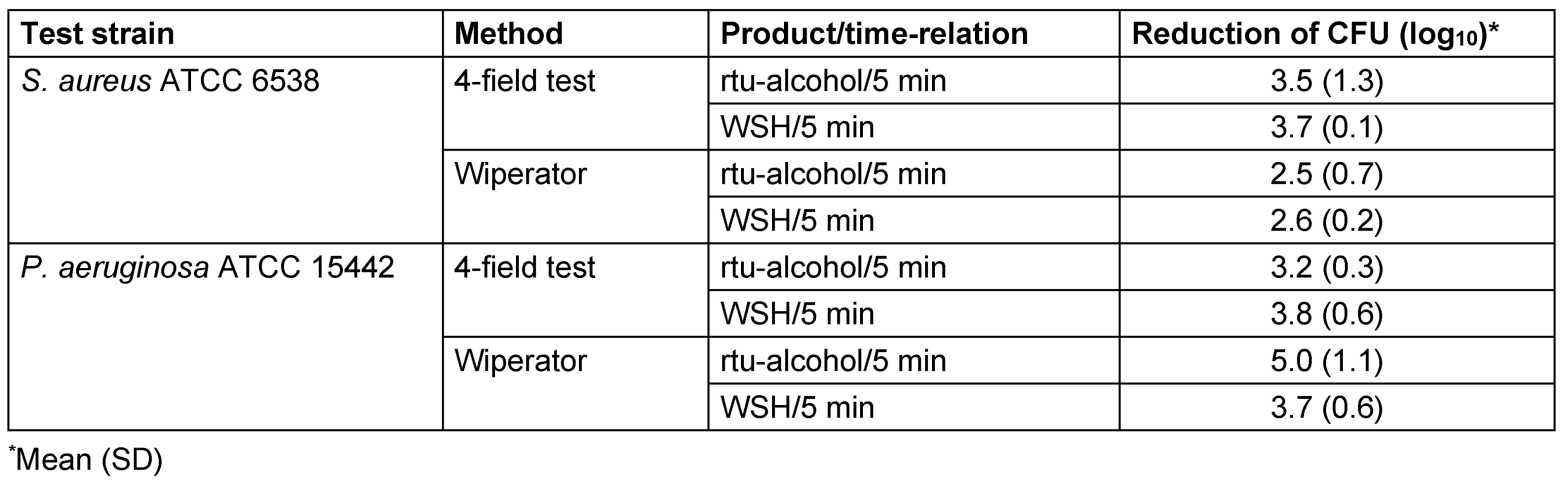
Mean log_10_ reduction of *S. aureus* and *P. aeruginosa* by exposure to an alcohol-based rtu-wipe with the 4-field test and Wiperator method at 5 min contact time (*n*=3)

**Table 6:**
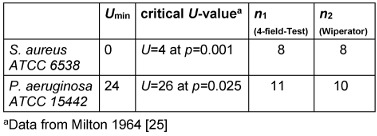
Test-statistics of Mann-Whitney-*U*-Test comparing log_10_ reduction of *S. aureus* and *P. aeruginosa* after exposition to WSH at 5 and 15 min with the 4-field test and Wiperator method

**Table 7:**
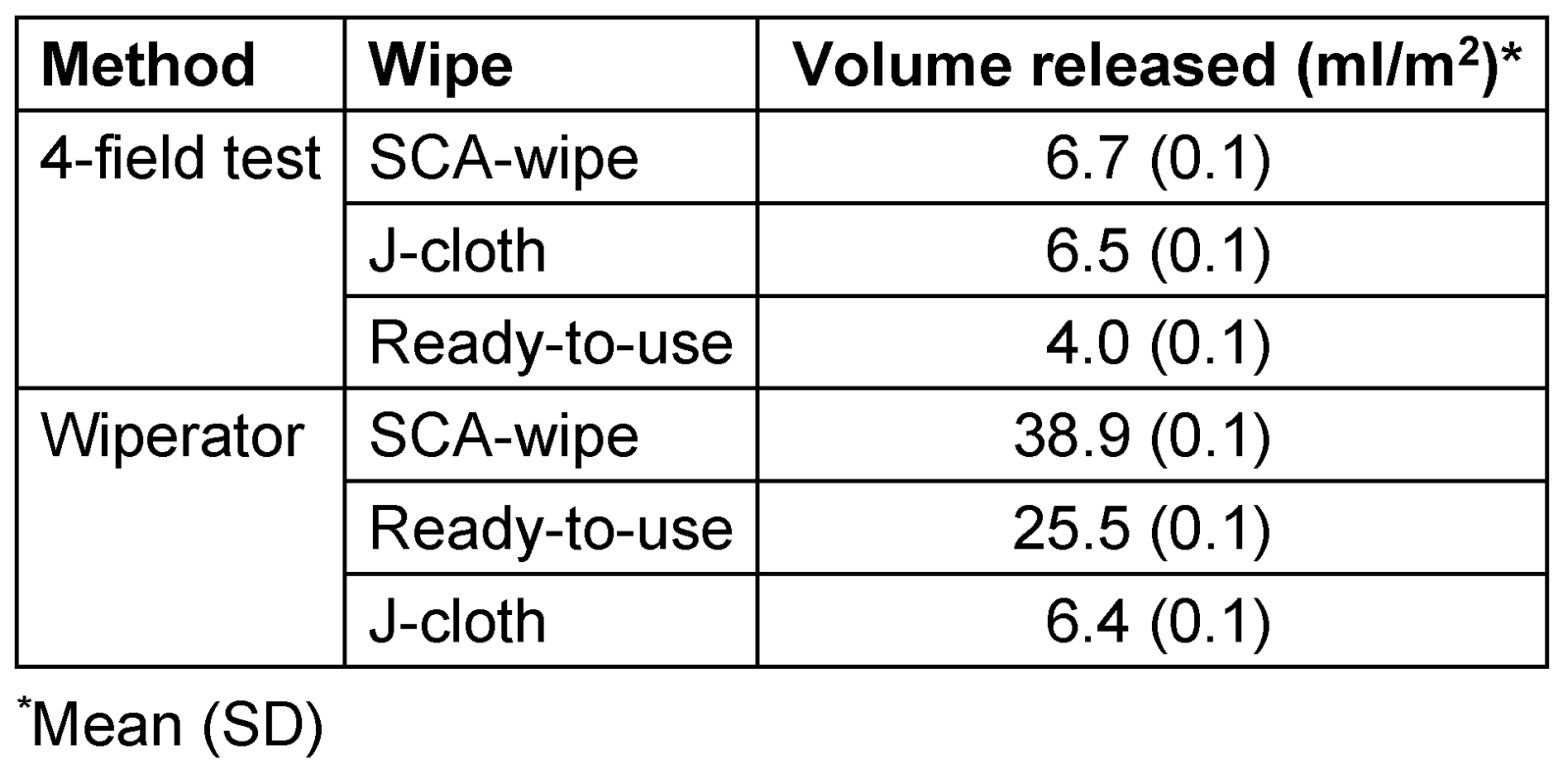
Volume output of J-cloth, SCA-wipe and rtu-wipe using the 4-field test and Wiperator method (*n*=3)
